# Microbubble assisted polyhydroxybutyrate production in *Escherichia coli*

**DOI:** 10.1186/s13104-016-2145-9

**Published:** 2016-07-09

**Authors:** Kadriye Inan, Fulya Ay Sal, Asif Rahman, Ryan J. Putman, Foster A. Agblevor, Charles D. Miller

**Affiliations:** Department of Molecular Biology and Genetics, Karadeniz Technical University, Trabzon, Turkey; Department of Biology, Karadeniz Technical University, Trabzon, Turkey; Bioengineering Branch, Space Biosciences Division, NASA Ames Research Center, Moffett Field, Ames, CA 94035-1000 USA; Universities Space Research Association, Mountain View, CA 94043 USA; Department of Biological Engineering, Utah State University, 4105 Old Main Hill, Logan, UT 84322-4105 USA

**Keywords:** Microbubble, Fermentation, Dissolved oxygen, Polyhydroxybutyrate, PHB

## Abstract

**Background:**

One of the potential limitations of large scale aerobic *Escherichia coli* fermentation is the need for increased dissolved oxygen for culture growth and bioproduct generation. As culture density increases the poor solubility of oxygen in water becomes one of the limiting factors for cell growth and product formation. A potential solution is to use a microbubble dispersion (MBD) generating device to reduce the diameter and increase the surface area of sparged bubbles in the fermentor. In this study, a recombinant *E. coli* strain was used to produce polyhydroxybutyrate (PHB) under conventional and MBD aerobic fermentation conditions.

**Results:**

In conventional fermentation operating at 350 rpm and 0.8 vvm air flow rate, an OD_600_ of 6.21 and PHB yield of 23 % (dry cell basis) was achieved. MBD fermentation with similar bioreactor operating parameters produced an OD_600_ of 8.17 and PHB yield of 43 % PHB, which was nearly double that of the conventional fermentation.

**Conclusions:**

This study demonstrated that using a MBD generator can increase oxygen mass transfer into the aqueous phase, increasing *E. coli* growth and bioproduct generation.

## Background

Continued use of petroleum based plastics is not sustainable, therefore alternative and environmentally friendly materials need to be developed. Biologically derived polymers could potentially be a candidate to replace petroleum based plastics in the near future [1]. Currently bioplastics make up only 1 % of the market share of all plastics used worldwide, in part due to various bottlenecks to making biological plastics economical on a large scale [2].

Polyhydroxyalkanoates (PHAs) are a class of biologically derived biopolymers with over 155 unique monomers [3]. The advantage of having many different monomers is that each possess different material characteristics and thus will be suitable for many potential applications [4]. One such PHA polymer is polyhydroxybutyrate (PHB), a short chain length (scl) polymer, which has a melting point and tensile strength similar to polypropylene and a Young’s modulus comparable to polystyrene [5]. PHB is naturally produced in organisms such as *Ralstonia eutropha*, *Alcaligenes latus, Burkholderia cepacia*, and *Bacillus sphaericus* [6–9]. The three genes responsible for PHB production *phb*A, *phb*B, *phb*C, can be recombinantly expressed in laboratory strains of *Escherichia coli* [10].

Large scale aerobic production of PHB can be carried out using genetically engineered *E. coli* strains in bioreactors [11]. The advantages of using recombinant *E. coli* for the production of PHB include rapid growth, accumulation of PHB greater than 50 % of cell weight [12], and the ability to utilize inexpensive carbon substrates [13, 14].

Previous studies have demonstrated that PHB production using recombinant systems such as *E. coli* have been hindered upon scaling up in part due to the use of large amounts of oxygen required for high bacterial growth and PHB generation. The amount of required oxygen supplied through increased agitation or supplemental oxygen fed into a traditional bioreactor are potentially cost prohibitive for PHB production upon scale-up [15–19]. One of the major challenges in many aerobic bioreactor setups is the poor solubility of oxygen in the media. The solubility of oxygen in water is approximately 0.217 mmol/l at 35 °C, and with the addition of salts and other media components the solubility is further reduced [20]. Additionally, high cell density cultures (HCDC) could also reduce the available dissolved oxygen in a culture at high OD’s [21].

A possible lower cost solution is to increase the relative surface area of air bubbles sparged into fermentor media. By reducing the bubble size, the oxygen transfer rate of the fermentor can be increased due to greater surface area. Furthermore, the velocity of the bubbles traveling from the bottom of a fermentor to the top is reduced, giving more time for oxygen transfer to the culture media. A microbubble dispersion (MBD) generator can reduce the size of a conventional sparged gas bubble from 3–5 mm to approximately 20–100 µm [20, 22]. Additionally, it has been suggested that microbubble sparged fermentors can be energy efficient up to 0.01 kW/m^3^ of fermentation capacity, providing lower overall operating costs [23].

Previous studies have utilized a MBD device in-line with a fermentor for recombinant product formation in *Pichia pastoris*, *Saccharomyces cerevisiae*, and *Trichoderma reesei* [20, 24, 25]. In the *P. pastoris* study, increase in cell growth and recombinant protein production were observed in the MBD system when compared to a standard sparged fermentation [24]. *Trichoderma reesei* was grown in a MBD system and demonstrated increase in cell mass [25]. Additionally, another study used a MBD system in-line with a 72 L fermentation vessel, demonstrating the versatile nature of the system for scale-up [26].

In this study, the objectives were to (1) demonstrate increased *E. coli* growth with a MBD system and (2) show increased PHB production from *E. coli* when MBD device was used compared to conventional air-sparged fermentation.

## Methods

### Strain selection

*E. coli* strain XL1-Blue (*recA1 endA1 gyrA96 thi*-*1 hsdR17 supE44 relA1 lac [F´ proAB lacIqZΔM15 Tn10 (TetR)]*) (Agilent Technologies, Santa Clara, CA) harboring the ampicillin resistant plasmid pBHR68 was used in all studies [10]. The XL1-Blue strain of *E. coli* was chosen for its ability to out-perform other *E. coli* strains for PHB production [27]. The plasmid pBHR68 was selected as it contained the lactose inducible phaCAB operon and had demonstrated PHB accumulation up to approximately 50 % of the dry cell weight after 48 h of growth in a minimal media [28].

### Culture media

For all experiments single colonies were picked from Luria–Bertani (LB) agar plates, inoculated in 5 mL LB media pre-cultures and grown overnight at 37 °C [29]. These 5 mL cultures were then used to start larger 50 mL cultures. Larger cultures consisted of a modified M9 minimal media containing: M9 salts (Na_2_HPO_4_, KH_2_PO_4_, NaCl, NH_4_Cl, Becton, Dickinson and Co, Sparks, MD), supplemented with 1.75 % (w/v) glucose (ACS grade, Acros Organics, Fair Lawn, NJ), 0.2 % (w/v) yeast extract (Becton, Dickinson and Co, Sparks, MD), and 100 µg/ml ampicillin (IBI Scientific, Peosta, IA). The addition of small amounts of yeast extract to the culture has been shown to increase PHB yields [27, 30]. The 50 mL culture was grown overnight in an orbital shaker table at 37 °C and 220 rpm and was used to seed 1 L fermentors of the same media composition. For PHB production studies using fermentors, 0.1 mM Isopropyl β-d-1-thiogalactopyranoside (IPTG) (Gold Biotechnology, Inc. St. Louis, MO) was added at the start of the fermentation (t = 0 h).

### Conventional air-sparged fermentation

BIOSTAT Q multi-fermentor bioreactor system (B. Braun Biotech International, Melsungen, Germany) was used with a 1 L working volume similar to that used in a previous study [31]. Conventional air-sparged culturing was conducted for the production of PHB at agitation rates of 350, 500, or 750 rpm with air-sparge rates of 0.4 or 0.8 vvm. The bioreactor was equipped with pH, dissolved oxygen (DO), and temperature probes. Both the DO and pH probes were calibrated after sterilization. The DO and pH of the media were not controlled but allowed to fall freely during fermentation. Turbidity (OD_600_) and glucose consumption were measured at 0, 4, 8, 12, 24, and 48 h. PHB production was measured at 12, 24, and 48 h. All experiments were duplicated.

### Microbubble dispersion sparged fermentation

A MBD generator was setup in-line with a 1 L BIOSTAT bioreactor running in batch-mode similar to that seen in a previous study [31]. In addition to bioreactor setup mentioned in the previous section, the MBD generator, containing a stainless steel disc 5 cm in diameter and 3 mm thick, was connected to a high-speed electrical motor that spun the disk at approximately 4000 rpm. Baffles 5 mm from the spinning disk generated a high sheared zone and air was fed into the MBD generator at 0.8 vvm to create microbubbles. Microbubbles were fed into the bioreactor using a peristaltic pump and Masterflex^®^ tubing at approximately 100-150 ml/min. A second peristaltic pump was used to pump fluid back to the MBD generator from the bioreactor at a similar flowrate to maintain a constant bioreactor volume. The recycling of the fermentation broth also served as a microbubble stabilizer because the natural surfactants generated by the *E. coli* assisted in stabilizing the microbubbles. As with the conventional air-sparged bioreactor, DO and pH were not controlled but allowed to fall freely. The media used in the MBD study was the same as that used in the air-sparge fermentation studies and no additional surfactants were used to stabilize the microbubbles generated. Turbidity (OD_600_), glucose consumption, and PHB production was measured at the same time points as conventional air-sparged fermentation experiments. Bioreactor impeller speed of 350 rpm was maintained over the course of the MBD study. All experiments were duplicated.

### Glucose analysis

Glucose concentration was determined with a glucose assay reagent kit (Sigma Aldrich, St Louis, MO) using a modified procedure similar to a previous study [13]. Briefly, 120 µl glucose assay reagent was added to 60 µl sample and incubated at 37 °C for 30 min. After incubation, 120 µl of 12 N H_2_SO_4_ was added to stop the enzymatic reaction. Absorbance was measured at 540 nm using a Synergy 2 microtiter plate reader (BioTek, Winooski, VT). Concentration calculations were carried out according to a glucose standard curve.

### PHB concentration determination

PHB concentration was determined by an NMR-GC correlation [32] at 12, 24, and 48 h respectively. The methods used in this study followed a procedure developed previously [32]. After fermentation approximately 100 mL of culture was centrifuged, frozen to −80 °C, and lyophilized. 15 mg of lyophilized sample was mixed with equal volumes of 5 % sodium hypochlorite and deuterated chloroform containing 0.03 % TMS (Cambridge Isotope Laboratories, Inc. Andover, MA). Samples were centrifuged to promote phase separation and the organic phase was analyzed using ^1^H NMR (Jeol ECX-300 NMR, Jeol USA, Inc. Peabody, MA). PHB concentration was determined from an NMR-GC calibration standard [32].

### Calculation of oxygen transfer coefficient

The volumetric oxygen transfer coefficient (k_L_a) was determined using a non-fermentative method (“gas out-gas in”) as discussed by Tribe et al. [33]. The advantage of this technique is that k_L_a can be determined directly from dissolved oxygen (DO) measurement [34]. Briefly, this method requires gassing the bioreactor with nitrogen to displace the dissolved O_2._ Next, air was gassed into the system at sparging rate of 0.4, 0.8 vvm, or via MBD. DO % was recorded over time until DO % reached approximately 90–95 %. Equation 1 [33] can be used for determining k_L_a, where: dC_L_/dt is change in DO over time (t), C* is saturated DO concentration, and C_L_ is DO concentration in the bioreactor.

1 $${\text{dC}}_{\text{L}} \;/\;{\text{dt}}\; = \;{\text{k}}_{\text{L}} {\text{a}}\;\left( {{\text{C}}^*\; - \;{\text{C}}_{\text{L}}} \right)$$

Integrating Eq. 1 and solving for k_L_a gives Eq. 2.

2 $${\text{k}}_{\text{L}} {\text{a}}\; = \;{ \ln }\;\left( {{\text{C}}^*\; - \;{\text{C}}_{\text{L}} } \right)\;/{\text{t}}$$

Plotting ln (C*−C_L_) verses time (t) produces a linear graph with a slope equal to the k_L_a in reciprocal time (h^−1^).

## Results and discussion

### *E. coli* growth using a MBD

**Fig. 1:**
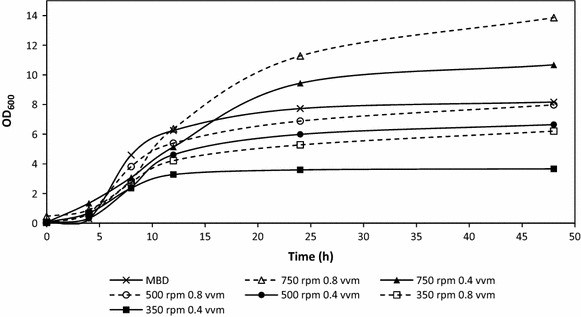
Growth profiles (OD_600_) for recombinant *E. coli* harboring the pBHR68 plasmid grown in conventional and MBD sparged fermentations at different agitation rates (n = 2)

The growth of XL1-Blue *E. coli* harboring the plasmid pBHR68 was found to be highest (OD_600_ approximately 13.86) in the bioreactor agitated at 750 rpm with an airflow of 0.8 vvm after 48 h post inoculation as shown in Fig. 1. The lowest growth was measured in the bioreactor operating at 350 rpm at 0.4 vvm yielding an average OD_600_ of 3.66. It was expected that the lowest agitation and air sparging rates would give the lowest overall growth due to oxygen limitations in the media. When the MBD generator was used, the average OD_600 _ of the culture was 8.17 at 48 h compared to 6.21 obtained for similar fermentation without MBD at 48 h post inoculation. This suggests that the presence of MBD aided in the growth of *E. coli* by increasing the amount of oxygen uptake by the bacteria.

It was observed that the faster the agitation speed (rpm), the shorter the time to reach saturated levels of DO concentration and thus the higher the volumetric oxygen transfer coefficient (k_L_a). The k_L_a for conventional air sparged and MBD systems are shown in Table 1. The k_L_a value increased from 16.81 to 23.47 h^−1^ for 0.4 vvm when the agitation rate was increased from 350 to 500 rpm. At 500 rpm doubling the aeration rate to 0.8 vvm showed only a slight improvement in k_L_a (27.53 h^−1^). At a higher agitation rate of 750 rpm with conventional air-sparging at 0.4 vvm the k_L_a achieved was 32.86 h^−1^. For the MBD system with a bioreactor operating at 350 rpm a k_L_a of 23.12 h^−1^ was observed, higher than the k_L_a of 22.01 h^−1^ observed in the bioreactor operating under similar conditions (350 rpm, 0.8 vvm). The k_L_a present in the MBD system was lower than the k_L_a in the 750 rpm systems but similar to the k_L_a in the 500 rpm air sparged studies, suggesting similar performance of the MBD system with the 500 rpm systems.

**Table 1:** Oxygen transfer coefficient *k*_L_ *a* (h^−1^) value for fermentations with conventional air sparged and MBD sparged fermentations (n = 2)

Air supply	Air-sparger	Air-sparger	Air-sparger	Air-sparger	Air-sparger	Air-sparger	MBD
Agitation rate (rpm)	350	350	500	500	750	750	350
Air sparge rate (vvm)	0.4	0.8	0.4	0.8	0.4	0.8	–
k_L_a (h^−1^)	16.81	22.01	23.47	27.53	32.86	34.29	23.12
OD_600_ (48 h)	3.66	6.21	5.99	7.98	10.68	13.86	8.17
PHB % (48 h)	20.25	23.93	26.12	46.46	47.33	54.08	43.14

**Fig. 2:**
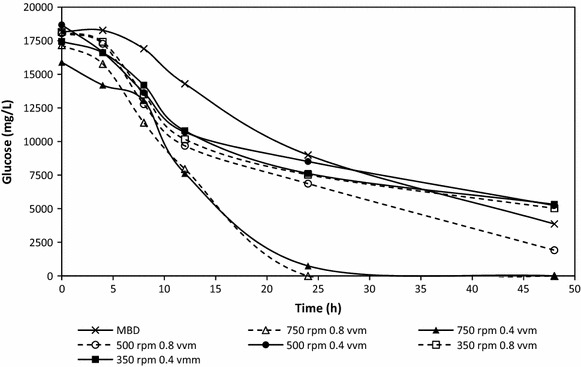
Glucose consumption (mg/L) over time (h) for PHB producing *E.coli* in conventional and MBD sparged fermentations at different agitation rates (n = 2)

As expected, glucose concentrations in the media decreased over the course of the 48 h study for all bioreactor conditions. Glucose was completely exhausted after approximately 24 h in bioreactor studies with an agitation rate of 750 rpm and sparging rate of 0.4 and 0.8 vvm. After 48 h glucose present in the MBD and 500 rpm 0.8 vvm studies approached 0 mg/L (Fig. 2). Since the initial measured concentration of glucose in all studies was approximately 1.75 % (w/v), coupling these findings with the k_L_a and OD_600_ results demonstrates that this system is oxygen limited.

**Fig. 3:**
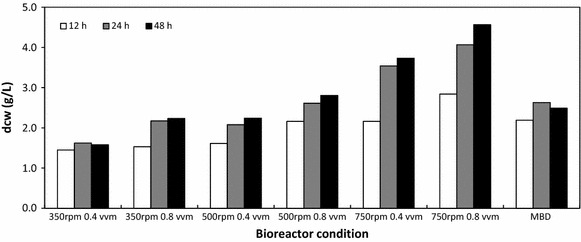
Dry cell weight (g/L) of recombinant *E. coli* harboring the pBHR68 plasmid grown in conventional and MBD sparged fermentations measured at 12, 24, and 48 h (n = 2)

Dry cell weight (dcw) correlated with *E. coli* growth measured with OD_600_. The most turbid culture (750 rpm, 0.8 vvm) also showed the highest dry cell weight after 48 h of culturing producing on average 4.57 g/L of dry biomass equivalent (Fig. 3). After 48 h the MBD system generated approximately 2.49 g/L dry cell weight, which was similar to the sparged system operating at 500 rpm and 0.8 vvm and higher than the sparged system operating at 350 rpm. The difference in dry cell weight between the bioreactor conditions can be attributed to the lack of DO present in the media. The theoretical amount of oxygen required for *E. coli* growing on glucose based media is approximately 1 g of oxygen for 1 g of *E. coli* biomass [35]. In this study the starting concentration of glucose was the same, yet in only two instances was the glucose completely exhausted, thus suggesting lack of usable oxygen in the system.

### PHB production analysis

**Fig. 4:**
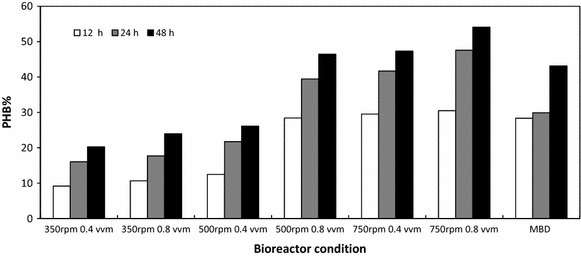
PHB production (% of dry cell weight) from recombinant *E. coli* grown in conventional and MBD sparged fermentations measured at 12, 24, and 48 h (n = 2)

All cultures showed increase in PHB accumulation through 12, 24, and 48 h respectively (Fig. 4). Although, as illustrated in Fig. 1, stationary phase was reached between 12 and 24 h for most experiments, bioreactors were run for 48 h as this allowed for accumulation of PHB. Previous studies have mentioned that PHB does not accumulate to significantly high levels during log phase of growth. Rather, acetyl-CoA is used for cell growth during exponential phase and then utilized for PHB production during the stationary phase, thus the motivation to culture for 48 h [36].

After 48 h of culturing, the average PHB % accumulation for conventional air-sparged experiments (0.4 vvm) at agitation rates of 350, 500, and 750 rpm, were 20.25, 26.12, and 47.33 %, respectively. At the same agitation rates with an increased air flow to 0.8 vvm the PHB % after 48 h was increased to 23.93, 46.46, and 54.08 %, respectively. For the 350 rpm bioreactor studies the difference between air sparged at 0.4 and 0.8 vvm did not provide a substantial increase in PHB production, whereas doubling the air flow rate for the 500 and 750 rpm agitation increased the PHB production significantly. The MBD bioreactor had an average PHB accumulation of 43.14 % 48 h post inoculation, which was similar to the 500 rpm agitation rate with 0.8 vvm air sparging and also outperformed the 350 rpm 0.8 vvm study. Accumulation of PHB to approximately 40–50 % after 48 h of culturing is similar to the results observed in shaker flask experiments in other studies with similar media composition and without pH or DO control [28, 30].

As discussed previously, oxygen limitations hindered both cell growth and PHB yields. This observation was also seen in prior studies, where oxygen limited fermentation experiments revealed reduced cell growth and lower PHB yields [37, 38]. Additionally, reduction in available oxygen could significantly affect native metabolic pathways in *E*. *coli*, including ATP generation, further limiting bacterial growth and hindering bioproduct generation [16]. In this study, the higher rpm experiments and MBD produced increased levels of PHB production compared to lower speed air-sparged fermentations, suggesting that oxygen limitations hindered cell growth and PHB generation.

Other fermentation based studies have used similar strains of XL1-Blue *E. coli* to produce PHB. One such study optimized media contents, maintained pH, used pure oxygen, and increased agitation speeds up to 1100 rpm. While this study produced high yields of PHB, the authors concluded that the scale-up of this system would not be cost effective due to considerable costs of using pure oxygen [27]. Combining a MBD approach with an optimized media used in the aforementioned study could potentially reduce the costs of bacterial fermentation to produce PHBs.

The results presented in this study provide justification for future work using the MBD generator in-line with a larger bioreactor for *E. coli* growth and PHB production. It has been reported that the power input per unit volume is approximately 1000 times higher in a laboratory based fermentation unit when compared to an industrial scale unit [20]. Scale-up from 1 to 50 L for *S. cerevisiae* fermentations using a MBD unit have been proven to be successful with a threefold reduction in power consumption using a MBD unit as compared to conventional air sparging [26]. It would be reasonable to suggest that *E. coli* grown in a 50 L MBD fed fermentor would give similar results. The next step would be to understand how the MBD unit would function upon scale-up and if a larger MBD system would be required for a larger scale fermentor. Furthermore, another consideration upon scale-up would be if any additional surfactants would be required to maintain microbubble size and if these additional surfactants affected *E.coli* growth and PHB yield.

## Conclusions

The production of PHB using genetically engineered *E. coli* requires a large oxygen supply that results in excessive energy consumption and therefore makes the process less economically competitive. The major challenge is the poor solubility of oxygen in the fermentation media because the amount of energy input required for agitation and aeration becomes exceedingly high. The use of a MBD generator can increase oxygen mass transfer into the aqueous phase, further promoting *E. coli* growth and bioproduct generation. The DO and pH of the media were not controlled in these experiments, though future work could consist of understanding the effects of controlling DO and pH on cell growth and PHB yield. Perhaps maintaining the pH at a stable level could have further increased the yield of PHB produced in system. Furthermore, developing an understanding how the MBD system would function in an industrial fed-batch environment could allow for this system be implemented in the future. Production of other recombinant products could also be tested using the methods outlined in this study.

## References

[1] Chanprateep S (2010). Current trends in biodegradable polyhydroxyalkanoates. J Biosci Bioeng.

[2] Sreedevi S, Unni K, Sajith S, Priji P, Josh M, Benjamin S (2015). Bioplastics: advances in polyhydroxybutyrate research. Advances in polymer science.

[3] Agnew DE, Pfleger BF (2013). Synthetic biology strategies for synthesizing polyhydroxyalkanoates from unrelated carbon sources. Chem Eng Sci.

[4] Rehm BHA (2010). Bacterial polymers: biosynthesis, modifications and applications. Nat Rev Microbiol.

[5] Khanna S, Srivastava AK (2005). Recent advances in microbial polyhydroxyalkanoates. Process Biochem.

[6] Ryu HW, Cho KS, Lee EG, Chang YK (2000). Recovery of poly(3-hydroxybutyrate) from coagulated Ralstonia eutropha using a chemical digestion method. Biotechnol Prog.

[7] Singhaboot P, Kaewkannetra P (2015). A higher in value biopolymer product of polyhydroxyalkanoates (PHAs) synthesized by *Alcaligenes latus* in batch/repeated batch fermentation processes of sugar cane juice. Ann Microbiol.

[8] Ramadas N, Sindhu R, Binod P, Pandey A (2013). Development of a novel solid-state fermentation strategy for the production of poly-3-hydroxybutyrate using polyurethane foams by *Bacillus sphaericus* NII 0838. Ann Microbiol.

[9] Dietrich D, Illman B, Crooks C (2013). Differential sensitivity of polyhydroxyalkanoate producing bacteria to fermentation inhibitors and comparison of polyhydroxybutyrate production from Burkholderia cepacia and Pseudomonas pseudoflava. BMC Research Notes.

[10] Spiekermann P, Rehm BHA, Kalscheuer R, Baumeister D, Steinbüchel A (1999). A sensitive, viable-colony staining method using Nile red for direct screening of bacteria that accumulate polyhydroxyalkanoic acids and other lipid storage compounds. Arch Microbiol.

[11] Ienczak JL, Schmidell W, De Aragão GMF (2013). High-cell-density culture strategies for polyhydroxyalkanoate production: a review. J Ind Microbiol Biotechnol.

[12] Wang F, Lee SY (1998). High cell density culture of metabolically engineered Escherichia coli for the production of poly(3-hydroxybutyrate) in a defined medium. Biotechnol Bioeng.

[13] Rahman A, Putman RJ, Inan K, Sal FA, Sathish A, Smith T, Nielsen C, Sims RC, Miller CD (2015). Polyhydroxybutyrate production using a wastewater microalgae based media. Algal Res.

[14] Rahman A, Anthony RJ, Sathish A, Sims RC, Miller CD (2014). Effects of wastewater microalgae harvesting methods on polyhydroxybutyrate production. Bioresour Technol.

[15] Choi JI, Lee SY (1997). Process analysis and economic evaluation for poly(3-hydroxybutyrate) production by fermentation. Bioprocess Eng.

[16] Lee SY, Choi J-i, Wong HH (1999). Recent advances in polyhydroxyalkanoate production by bacterial fermentation: mini-review. Int J Biol Macromol.

[17] Lee SY (1996). High cell-density culture of *Escherichia coli*. Trends Biotechnol.

[18] Choi J, Lee SY (1999). Factors affecting the economics of polyhydroxyalkanoate production by bacterial fermentation. Appl Microbiol Biotechnol.

[19] Van Wegen RJ, Ling Y, Middelberg APJ (1998). Industrial production of polyhydroxyalkanoates using *Escherichia Co*li: an economic analysis. Chem Eng Res Des.

[20] Kaster J, Michelsen D, Velander W (1990). Increased oxygen transfer in a yeast fermentation using a microbubble dispersion. Appl Biochem Biotechnol.

[21] Choi JH, Keum KC, Lee SY (2006). Production of recombinant proteins by high cell density culture of *Escherichia coli*. Chem Eng Sci.

[22] Sebba F (1985). An improved generator for micron-sized bubbles. Chem Ind.

[23] Bredwell MD, Worden RM (1998). Mass-transfer properties of microbubbles. 1. experimental studies. Biotechnol Prog.

[24] Zhang W, Li ZJ, Agblevor FA (2005). Microbubble fermentation of recombinant *Pichia pastoris* for human serum albumin production. Process Biochem.

[25] Weber J, Agblevor FA (2005). Microbubble fermentation of *Trichoderma reesei* for cellulase production. Process Biochem.

[26] Hensirisak P, Parasukulsatid P, Agblevor FA, Cundiff JS, Velander WH (2002). Scale-up of microbubble dispersion generator for aerobic fermentation. Appl Biochem Biotechnol.

[27] Lee SY, Chang HN (1995). Production of poly(3-hydroxybutyric acid) by recombinant *Escherichia coli* strains: genetic and fermentation studies. Can J Microbiol.

[28] Rahman A, Linton E, Hatch AD, Sims RC, Miller CD (2013). Secretion of polyhydroxybutyrate in *Escherichia coli* using a synthetic biological engineering approach. J Biol Eng.

[29] Sambrook J, Russel DW (2001). Molecular cloning: a laboratory manual.

[30] Kang Z, Wang Q, Zhang HJ, Qi QS (2008). Construction of a stress-induced system in *Escherichia coli* for efficient polyhydroxyalkanoates production. Appl Microbiol Biotechnol.

[31] Zhao B, Agblevor FA, Ritesh KC, Jelesko JG (2013). Enhanced production of the alkaloid nicotine in hairy root cultures of *Nicotiana tabacum* L. Plant Cell, Tissue and Organ Culture (PCTOC).

[32] Linton E, Rahman A, Viamajala S, Sims RC, Miller CD (2012). Polyhydroxyalkanoate quantification in organic wastes and pure cultures using a single-step extraction and 1H NMR analysis. Water Sci Technol.

[33] Tribe LA, Briens CL, Margaritis A (1995). Determination of the volumetric mass transfer coefficient (kLa) using the dynamic “gas out–gas in” method: analysis of errors caused by dissolved oxygen probes. Biotechnol Bioeng.

[34] Bandyopadhyay B, Humphrey AE, Taguchi H (1967). Dynamic measurement of the volumetric oxygen transfer coefficient in fermentation systems. Biotechnol Bioeng.

[35] Shiloach J, Fass R (2005). Growing *E. coli* to high cell density—a historical perspective on method development. Biotechnol Adv.

[36] Lee SY, Yim KS, Chang HN, Chang YK (1994). Construction of plasmids, estimation of plasmid stability, and use of stable plasmids for the production of poly(3-hydroxybutyric acid) by recombinant *Escherichia coli*. J Biotechnol.

[37] Wang FL, Lee SY (1997). Production of poly(3-hydroxybutyrate) by fed-batch culture of filamentation-suppressed recombinant *Escherichia coli*. Appl Environ Microbiol.

[38] de Almeida A, Giordano AM, Nikel PI, Pettinari MJ (2010). Effects of aeration on the synthesis of Poly(3-Hydroxybutyrate) from glycerol and glucose in recombinant *Escherichia coli*. Appl Environ Microbiol.

